# Ras oncogene mutations and survival in patients with lung cancer

**DOI:** 10.1038/sj.bjc.6603112

**Published:** 2006-04-25

**Authors:** G Ferretti, A Felici, F Cognetti

**Affiliations:** 1 Department of Medical Oncology, Regina Elena Cancer Institute, Via Elio Chianesi 53, Rome 00144, Italy

**Sir,**

The proto-oncogene ras is mutated in 20% of lung cancer (Mascaux *et al*, 2005). Among 36 patients with primary operable non-small-cell lung cancer (NSCLC), we detected seven (19.4%) with K-ras exon I mutations on tumour samples (Ferretti *et al*, 2000). However, the prognostic significance of ras for survival in this disease remains controversial.

Aggregating data obtained by univariate analysis in retrospective trials, ras gene alteration appears to be a poor prognostic factor for survival in NSCLC (Mascaux *et al*, 2005). The authors concluded that ras appears to be a pejorative prognostic factor in terms of survival in NSCLC globally, in adenocarcinomas and when it is studied by polymerase chain reaction. In 44 patients with stage III NSCLC undergoing tumour resection after neoadjuvant treatment, the presence of a ras mutation, which was found independently of gender, age, tumour stage and clinical response status, was a significant predictor for a poor progression-free survival even after complete resection (Broermann *et al*, 2002). Patients with K-ras mutant NSCLC showed poorer clinical outcomes when treated with erlotinib and chemotherapy (Eberhard *et al*, 2005). Furthermore, in NSCLCs with ras mutation, the overexpression of Skp2, a protein that plays a critical role in cell cycle progression, was a significant independent poor prognostic marker of survival (Zhu *et al*, 2004).

By contrast, in the adjuvant setting, among 227 patients with surgically resected NSCLC, the K-ras mutations (codons 1–31) were not predictive markers of a shorter survival (Moldvay *et al*, 2000). Other authors had previously shown only a trend toward improved survival for patients with wild-type ras compared with those with mutant ras who were randomised to receive adjuvant postoperative radiotherapy or radiotherapy plus concurrent chemotherapy (Schiller *et al*, 2001). At the multivariate analysis, a trend bordering on statistical significance for K-ras was registered as well. However, according to Winton *et al* (2005), in patients with completely resected stage IB–II NSCLC, adjuvant chemotherapy did not confer a survival advantage in those whose tumours had ras mutations, whereas it did in patients with wild-type ras. It must be highlighted that in this study the interaction analysis showed that the effect of ras mutations on the treatment outcome was not statistically significant.

Thus, in order to stratify variables for future treatment approaches, the prognostic and predictive impact of ras mutation status still needs to be confirmed in prospective trials with appropriate multivariate analysis.
